# PANoptosis: a novel therapeutic target and mechanistic insight into traditional Chinese medicine intervention in inflammatory bowel disease: a review

**DOI:** 10.3389/fphar.2026.1880406

**Published:** 2026-10-13

**Authors:** Xiaojian Li, Wei Guan, Yongduo Yu, Xinyao Xu, Shujian An

**Affiliations:** 1 Liaoning University of Traditional Chinese Medicine, Shenyang, China; 2 Department of Hemorrhoids and Fistula I, The Third Affiliated Hospital of Liaoning University of Traditional Chinese Medicine, Shenyang, China; 3 Department of Proctology, The Second Affiliated Hospital of Liaoning University of Traditional Chinese Medicine, Shenyang, China

**Keywords:** inflammatory bowel disease, intestinal barrier, PANoptosis, programmed cell death, traditional Chinese medicine

## Abstract

The rising incidence of inflammatory bowel disease (IBD) and the limited efficacy of current therapies underscore the need for novel strategies. PANoptosis is an inflammatory programmed cell-death process integrating molecular features of pyroptosis, apoptosis, and necroptosis. Emerging evidence suggests that PANoptosis-related genes and signaling nodes may contribute to intestinal epithelial injury, mucosal immune activation, barrier disruption, and inflammatory amplification in inflammatory bowel disease. Traditional Chinese medicine and its active constituents have been reported to regulate inflammation, intestinal barrier integrity, gut microbiota, and several cell-death pathways. However, direct evidence that these agents inhibit *bona fide* PANoptosis in intestinal models remains limited. Current mechanistic evidence is strongest for selected compounds, such as baicalin, which has been shown in macrophages to interfere with mitochondrial Z-DNA formation and ZBP1-PANoptosome assembly, whereas other proposed mechanisms are largely extrapolated from extra-intestinal models or from studies of apoptosis, pyroptosis, necroptosis, and inflammatory signaling. This narrative review summarizes the molecular basis of PANoptosis in IBD and critically evaluates the potential of traditional Chinese medicine-derived compounds and formulas to modulate upstream sensors, adaptor proteins, and downstream effectors of PANoptosis-related pathways. We emphasize that future studies should verify these mechanisms in intestinal epithelial cells, intestinal organoids, DSS/TNBS/IL-10-deficient colitis models, and human IBD tissues.

## Graphical Abstract

**Figure:**
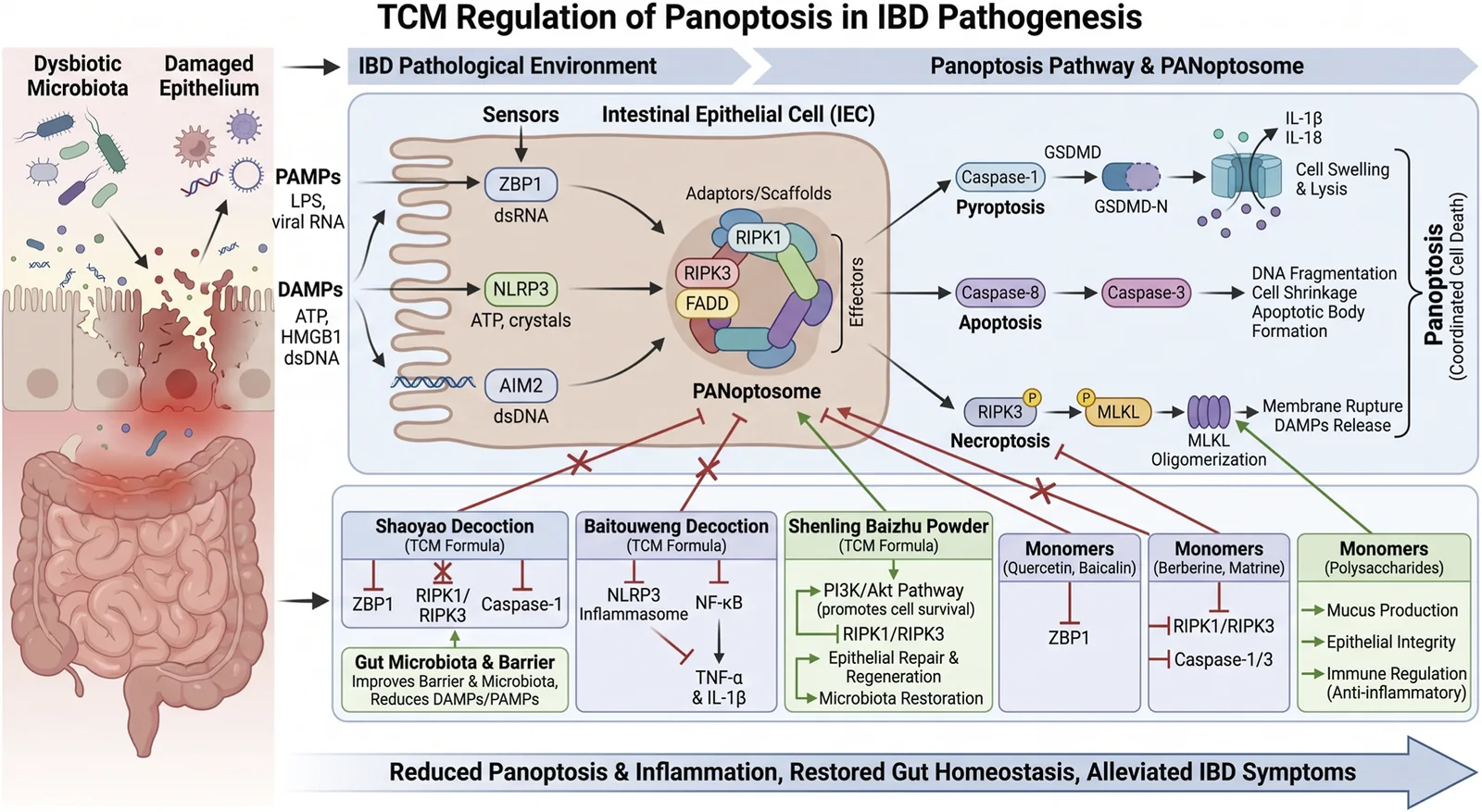

## 1 Introduction

Inflammatory Bowel Disease (IBD) represents a group of chronic, relapsing, multifactorial intestinal inflammatory disorders, primarily encompassing Ulcerative Colitis (UC) and Crohn’s Disease (CD). Globally, the incidence and prevalence of IBD are steadily rising, particularly in industrialized nations, establishing it as a significant public health challenge (Kaplan, 2025; Prioritizing inflammatory bowel disease, 2025). Despite remarkable advances in modern medicine, IBD remains a refractory disease. Its core pathological hallmark is an aberrant immune response to gut microbiota dysbiosis, which leads to disruption of the intestinal barrier, sustained inflammation, and tissue damage (Han et al., 2025; Lin et al., 2026). Conventional therapeutic strategies, such as aminosalicylates, glucocorticoids, and immunosuppressants, provide disease control in a subset of patients to some extent, yet they are often constrained by limited efficacy, pronounced adverse effects, and waning effectiveness upon long-term administration. In recent years, biologics targeting tumor necrosis factor-α (TNF-α) have offered new hope for patients with moderate-to-severe IBD. However, a substantial proportion of patients still exhibit primary non-response or develop secondary loss of response to existing biologics, underscoring the pressing need to identify novel therapeutic targets and develop innovative strategies (Vieujean et al., 2022; Puca et al., 2024; Estevinho and Noor, 2026).

The conceptualization of “PANoptosis” offers a novel perspective for deciphering the intricate pathophysiological processes of IBD. PANoptosis is not merely the concurrent or mixed occurrence of apoptosis, pyroptosis, and necroptosis—the three recognized modalities of programmed cell death. Instead, it is an integrated, highly coordinated modality of cell death driven synergistically by a large multi-protein complex termed the PANoptosome under specific pathological conditions (Lee et al., 2021). The assembly of the PANoptosome results from the integration of multiple signaling cascades, and its activation can simultaneously trigger or modulate the aforementioned three, and potentially more, cell death pathways, thereby efficiently eliminating damaged or infected cells in a short time frame. This complex regulatory network positions PANoptosis as a critical hub that interconnects cellular metabolism, stress responses, and innate immunity, playing an indispensable role in maintaining tissue homeostasis and counteracting pathogen invasion. Therefore, an in-depth exploration of the molecular mechanisms of PANoptosis and its specific roles in IBD holds inestimable value for unraveling disease pathogenesis and identifying breakthrough therapeutic targets (Zhang et al., 2025b; Triantafillidis and Karakatsanis, 2026).

Traditional Chinese Medicine (TCM), as a treasured heritage of the Chinese nation, has accumulated a wealth of theoretical and practical experience over millennia, demonstrating unique advantages notably in regulating the holistic balance of the organism. One of the core tenets of TCM is the “holistic concept” and “syndrome differentiation and treatment,” which emphasizes restoring the body’s dynamic internal equilibrium through multi-component, multi-target, and multi-pathway synergistic actions. This “multi-target” regulatory characteristic endows TCM with an inherent compatibility and significant potential for application when confronting complex biological events like PANoptosis, which involves numerous signaling pathways and a multitude of protein molecules. A substantial body of research has demonstrated that Chinese herbal medicines and their active constituents can effectively modulate inflammatory responses, safeguard the intestinal mucosal barrier, regulate the gut microbiota, and ultimately alleviate IBD symptoms (Wang et al., 2024). This narrative review integrates current molecular evidence implicating PANoptosis in IBD pathogenesis and critically discusses the potential multi-target regulatory mechanisms by which selected herbal monomers and classical traditional Chinese medicine formulas may influence the PANoptosome-related signaling network. Rather than providing a formal systematic review or meta-analysis, this article aims to synthesize mechanistic evidence, identify translational gaps, and propose testable hypotheses for PANoptosis-targeted IBD therapies based on ethnopharmacological principles (Liang et al., 2024). The classification of cell death-related proteins is shown in Table 1.

**TABLE 1:** Key proteins involved in PANoptosis and their classification.

Classification of pro-apoptotic proteins	Representative protein	Major function	Role in PANoptosis	Key reference(s)
Sensor	ZBP1, NLRP3, AIM2	Detect pathogenic or injury signals within the cell (such as dsRNA, dsDNA, ATP, etc.) as initiators of the PANoptotic pathway.	In the IBD environment, the identification of DAMPs or viral products resulting from gut microbiota dysbiosis triggers the formation of PANoptosomes.	Wang et al., 2024
Adapter protein	RIPK1, RIPK3, FADD	As a signaling hub, it bridges upstream sensor proteins and downstream effector proteins to form oligomers or protein complexes, thereby transmitting and amplifying death signals.	In PANoptosome, it acts as the “commander,” determining the direction of signaling flow and directing cells into apoptosis, necroptosis, or pyroptosis pathways, or facilitating switching between these pathways.	Liang et al., 2024
Effect protein	Caspase-1/3/8, MLKL	Directly initiates the cell death program. Caspase-1 cleaves GSDMD, triggering pyroptosis; Caspase-3 mediates apoptosis; the RIPK3-MLKL pathway induces necroptosis.	Upon formation of the PANoptosome, the effector proteins are activated, collectively leading to cell membrane rupture and release of cellular contents. This constitutes the direct cause of intestinal barrier disruption and exacerbation of inflammation in IBD.	Lee et al., 2021

## 2 Literature search and evidence synthesis strategy

This article is a narrative review. A structured literature search was conducted to identify representative studies related to PANoptosis, inflammatory bowel disease, intestinal epithelial cell death, intestinal barrier dysfunction, gut microbiota dysbiosis, and traditional Chinese medicine or natural products. Searches were performed in PubMed, Web of Science, Scopus, Embase, China National Knowledge Infrastructure, Wanfang, VIP, and Google Scholar using combinations of the following terms: “PANoptosis”, “PANoptosome”, “ZBP1”, “RIPK1”, “RIPK3”, “caspase”, “gasdermin”, “pyroptosis”, “apoptosis”, “necroptosis”, “inflammatory bowel disease”, “ulcerative colitis”, “Crohn’s disease”, “intestinal barrier”, “gut microbiota”, “traditional Chinese medicine”, “Chinese herbal medicine”, “flavonoid”, “alkaloid”, “polysaccharide”, and the names of representative compounds and formulas.

Because the purpose of this manuscript is to provide a mechanistic and conceptual synthesis rather than a systematic review, no PICOS framework, PRISMA flow diagram, formal study-selection count, or risk-of-bias assessment was performed. Priority was given to studies directly examining PANoptosis-related molecules or pathways. Where evidence was obtained from non-intestinal disease models, this has been explicitly indicated in the text and tables, and mechanistic extrapolations to IBD have been presented as hypotheses rather than established conclusions.

## 3 The molecular landscape of PANoptosis: synergy and switching mechanisms among three programmed cell death pathways

PANoptosis is frequently initiated by the integrated sensing of DAMPs and PAMPs. In IBD, luminal PAMPs such as lipopolysaccharide, peptidoglycan, and flagellin, together with DAMPs including HMGB1, ATP, mtDNA, and extracellular histones, activate innate immune receptors such as TLRs, NOD-like receptors, and ZBP1. These signals converge on shared hubs including RIPK1/RIPK3, CASP8, NLRP3, and gasdermins, leading to coordinated activation of apoptosis, pyroptosis, and necroptosis. This synergistic sensing mechanism may explain why epithelial injury in IBD often evolves into self-amplifying inflammatory cell death.

The molecular basis of PANoptosis constitutes a precise and intricate network, where the core lies in the tight coordination and reciprocal switching between distinct programmed cell death (PCD) pathways. This process is not a simple linear cascade but a dynamically regulated system composed of multiple sensor, adaptor, and effector proteins (Sun et al., 2025). Understanding these key molecules and their interactions is a prerequisite for elucidating how PANoptosis is initiated and executes its biological functions (Wang L. et al., 2023).

The initiation of PANoptosis relies on a series of “sensor” proteins that function as sentinels, responsible for detecting diverse danger signals of both intracellular and extracellular origin, such as pathogen-associated molecular patterns (PAMPs), damage-associated molecular patterns (DAMPs), or cellular stress (Qi et al., 2023). These sensor proteins primarily include Z-DNA binding protein 1 (ZBP1), NLR family pyrin domain-containing 3 (NLRP3), and AIM2-like receptors (AIM2) (Lee et al., 2021; Upmanyu and Upadhyay, 2025). ZBP1 recognizes abnormal double-stranded RNA of viral or host provenance; NLRP3 predominantly responds to a variety of endogenous and exogenous stimuli, such as ATP, uric acid crystals, and viral infection; AIM2 specifically detects double-stranded DNA within the cytoplasm (Hao et al., 2022; DeAntoneo et al., 2023). Upon sensing these signals, these sensor proteins undergo conformational changes, exposing specific interaction domains, thereby recruiting downstream adaptor proteins and initiating the subsequent cascade.

Adaptor/scaffold proteins, such as receptor-interacting serine/threonine-protein kinase 1 (RIPK1), RIPK3, and Fas-associated death domain protein (FADD), act as critical hubs linking upstream sensors to downstream effector proteins (Green et al., 2011). They do not directly execute cell death but instead converge and relay distinct signaling pathways by forming oligomers or multi-protein complexes. For instance, within the necroptosis pathway, activated RIPK1 recruits and phosphorylates RIPK3, and together they assemble into the so-called “necrosome.” In the apoptosis pathway, FADD serves as a bridge, connecting death receptors to Caspase-8 to initiate the apoptotic proteolytic cascade (Wegner et al., 2017). On the grand stage of PANoptosis, these adaptor proteins play the role of “conductors,” dictating the directional flow of signaling pathways.

Effector proteins are the ultimate “executioners” that carry out the cell death sentence, primarily encompassing the cysteine-aspartic protease (Caspase) family and mixed lineage kinase domain-like protein (MLKL). Among them, Caspase-1, Caspase-3, and Caspase-8 play central roles in their respective pathways (Ai et al., 2024). The activation of Caspase-1 is a hallmark event of pyroptosis; it cleaves and activates Gasdermin D (GSDMD), leading to pore formation in the plasma membrane and culminating in inflammatory cell death (He et al., 2015; Kayagaki et al., 2015; Shi et al., 2015). Caspase-3 is the terminal executioner protease of the apoptosis pathway, responsible for cleaving numerous substrate proteins and leading to complete cellular disassembly (Fernandes-Alnemri et al., 1994; Nadendla et al., 2025). RIPK3 phosphorylates MLKL, inducing its oligomerization and translocation to the plasma membrane, similarly causing loss of membrane integrity and mediating necroptosis (Meng et al., 2021). The existence of these effector proteins ensures that, once initiated, the cell death program proceeds irreversibly to completion.

Complex synergistic and switching mechanisms exist among these three major PANoptosis pathways. Within the pathological microenvironment of IBD, the stress and signaling milieus to which cells are exposed change rapidly, rendering a single mode of cell death often insufficient to cope with the intricate challenges (Gullett et al., 2022; Zhang et al., 2025b). Consequently, cells have evolved an exquisite mechanism that permits flexible switching between different death pathways based on signal intensity and type. For example, under certain conditions, RIPK1 can recruit either FADD or RIPK3, thereby oscillating between the apoptosis and necroptosis pathways (Orozco et al., 2014). Activation of ZBP1 can not only induce necroptosis but also promote apoptosis, and, under specific circumstances, synergize with NLRP3 to amplify the pyroptotic response (Malireddi et al., 2019; Oh and Lee, 2023). This cross-talk and signal diversion among pathways form the molecular basis of the PANoptosome. When multiple sensors are concurrently activated, they can jointly recruit adaptor proteins such as RIPK1, RIPK3, and FADD to form a larger, more stable PANoptosome complex. This complex can simultaneously accommodate the activation modules of apoptosis, pyroptosis, and necroptosis, leading to the three death modalities occurring almost synchronously or sequentially within the same cell—this constitutes the essence of PANoptosis (Samir et al., 2020; Gullett et al., 2022). This integrated cell death modality is highly efficient at clearing severely impaired or infected cells, yet it is precisely its uncontrolled activation that may lead to severe tissue damage and uncontrolled inflammation in IBD. Hence, precisely understanding and therapeutically intervening in the molecular interaction network is pivotal for developing novel IBD therapies (Zhang et al., 2026; Figure 1).

**FIGURE 1:**
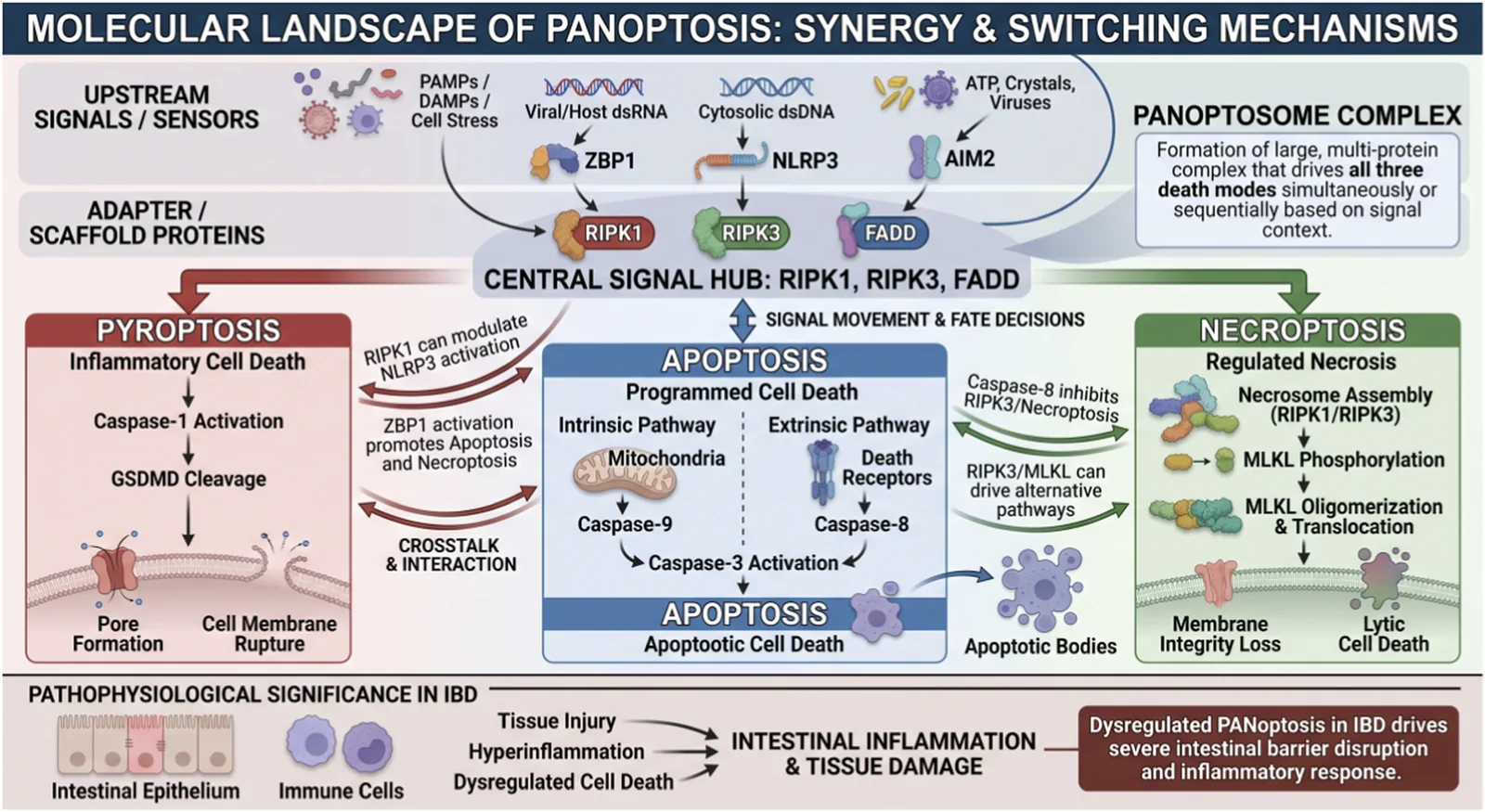
Molecular landscape of PANoptosis: Synergy and switching mechanisms.

## 4 The dual role of PANoptosis in IBD pathophysiology

PANoptosis plays a paradoxical dual role in the onset and progression of Inflammatory Bowel Disease (IBD). On the one hand, it serves as an intrinsic defense mechanism aimed at eliminating damaged, senescent, or pathogen-infected cells, thereby protecting the host from further insults (Aufschnaiter et al., 2026). On the other hand, when excessively activated, PANoptosis transforms into a “destroyer” of self-tissues, actively fueling the pathological progression of IBD (Zhao and Lin, 2024; Dong et al., 2025). This duality is primarily manifested in two key aspects: the disruption of intestinal barrier integrity and severe perturbation of the immune system.

### 4.1 Disruption of intestinal barrier integrity

PANoptosis is a key driving force behind the breakdown of the intestinal barrier in IBD patients. The intestinal epithelial cell layer constitutes the first physical line of defense of the gut, and its integrity is indispensable for preventing the translocation of noxious substances and pathogens into the bloodstream (Arumugam et al., 2025; Selvakumar and Samsudin, 2025). Under the pathological conditions of IBD, various endogenous and exogenous stimuli (e.g., gut microbiota dysbiosis, ischemia-reperfusion injury, toxins) trigger intense stress responses in intestinal epithelial cells, subsequently instigating the activation of the PANoptosis pathway (Lu et al., 2024; Tan et al., 2025). When PANoptosis is initiated on a large scale, a massive number of intestinal epithelial cells undergo rapid death and desquamation, resulting in widespread mucosal erosions and ulcerations (Wang J.M. et al., 2023; Liu C. et al., 2024). This rapid cell loss not only directly compromises the physical integrity of the intestinal barrier but also disrupts intercellular tight junctions, thereby increasing intestinal permeability, a condition known as “leaky gut.” This state allows the gut microbiota and its components (e.g., lipopolysaccharide, LPS), normally confined within the intestinal lumen, to traverse the epithelial barrier and infiltrate the lamina propria and systemic circulation, thereby inducing and exacerbating both systemic and local inflammatory cascades (Caballero-Mateos et al., 2025; Selvakumar and Samsudin, 2025). Therefore, excessive PANoptosis constitutes a central pathological basis for intestinal barrier dysfunction and tissue destruction in IBD.

### 4.2 Perturbation of the immune system

Activation of PANoptosis is intimately linked to immune dysregulation, as it escalates localized cell death events into a devastating “cytokine storm” through the massive release of damage-associated molecular patterns (DAMPs). When cells undergo pyroptosis or necroptosis, pores form in their plasma membranes, leading to the leakage of intracellular contents, including DAMPs, into the extracellular milieu (Kappelhoff et al., 2024; Liu Y. et al., 2024; Wright et al., 2025). These DAMPs, such as high mobility group box 1 (HMGB1), ATP, uric acid crystals, and specific cytokines like IL-1β, are recognized by pattern recognition receptors (PRRs) on the surface of adjacent immune cells (particularly macrophages and dendritic cells). This immediately activates these innate immune cells, inducing them to secrete copious amounts of pro-inflammatory cytokines (e.g., TNF-α, IL-6, IL-1β) and chemokines, which in turn recruit more immune cells (e.g., neutrophils, monocytes) to the inflammatory site. This cascading amplification of the inflammatory response establishes a positive feedback loop: excessive PANoptosis → massive DAMP release → activation of innate immune cells like macrophages → eruption of a cytokine storm → further induction of PANoptosis in more cells (Pandian and Kanneganti, 2022; Review on the Mechanism of and Therapies Targeting PANoptosis in Ulcerative Colitis, n. d.). This vicious cycle is one of the fundamental causes of the relapsing nature of IBD. Studies have demonstrated that in the inflamed intestinal tissues of IBD patients, the expression levels of PANoptosis-associated genes are significantly upregulated, and this correlates positively with the density of infiltrating immune cells and local cytokine concentrations (Zhang et al., 2025a; Zhao M. et al., 2026; Zhao Y. et al., 2026; Figure 2).

**FIGURE 2:**
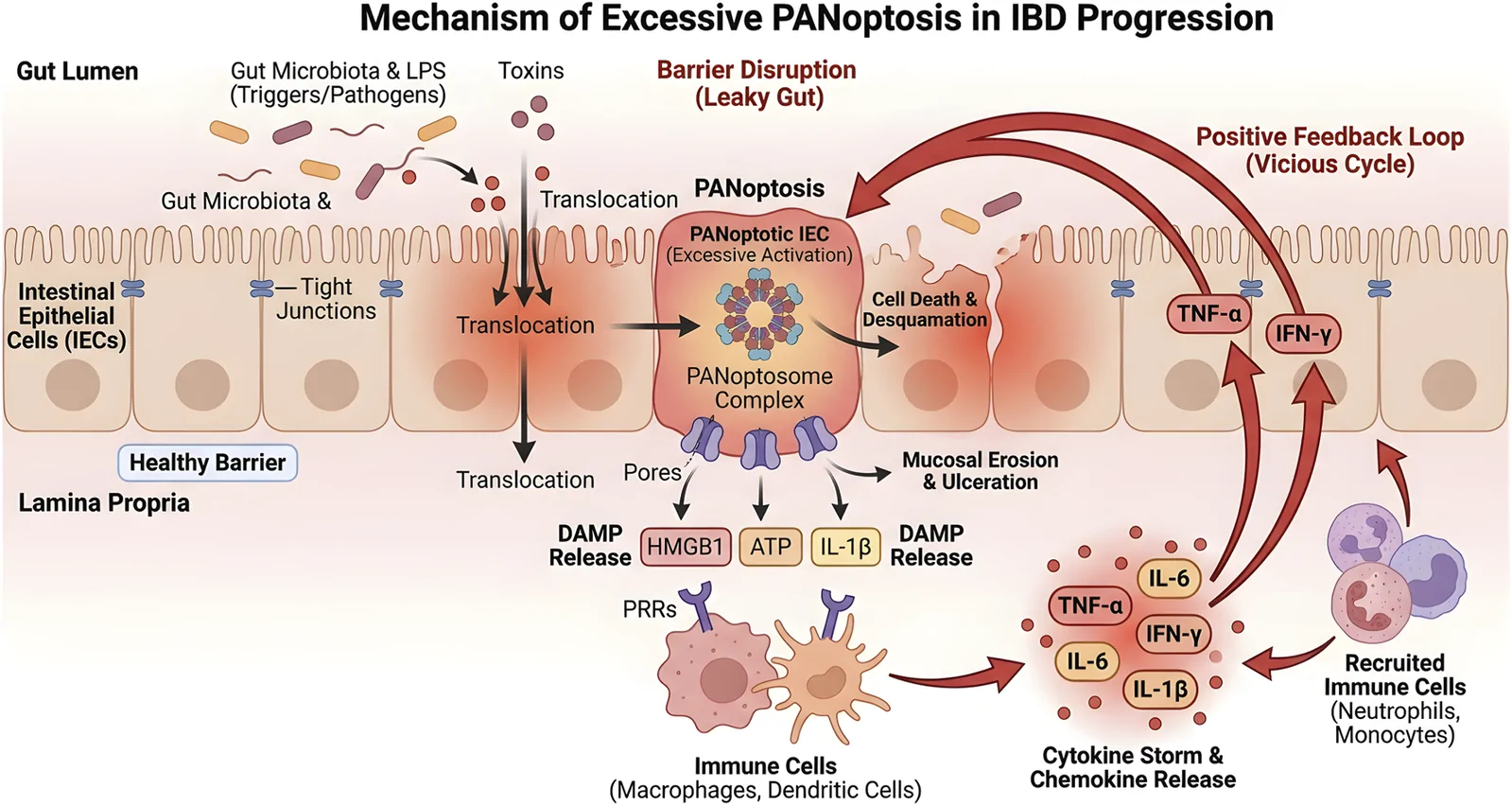
Dual role of PANoptosis in IBD pathophysiology: defense vs. destruction.

Furthermore, a close mutual interaction exists between the gut microbiome and PANoptosis. Gut microbiota dysbiosis is considered a key triggering factor in the pathogenesis of IBD. A dysbiotic microbial community can instigate PANoptosis in host cells through multiple mechanisms. For example, certain pathogenic bacteria can directly secrete toxins (e.g., Shiga toxin) or activate the NLRP3 inflammasome within intestinal epithelial cells via alternative mechanisms, thereby initiating pyroptosis (Fox et al., 2020; Wright et al., 2024; Li et al., 2025; Ricci-Azevedo et al., 2026). Other studies have found that dysbiosis-induced alterations in the proportions of short-chain fatty acids (SCFAs), particularly a reduction in butyrate, can affect the activity of histone deacetylases (HDACs), thereby modulating the expression of genes associated with cell death and potentially promoting PANoptosis indirectly (Hinrichsen et al., 2021; Yang et al., 2026). Concurrently, bacterial components such as LPS can activate the RIPK1/RIPK3-dependent necroptosis pathway through Toll-like receptor 4 (TLR4) signaling (Cao et al., 2018; Lim et al., 2019). This excessive host cell death, triggered by gut microbiota dysbiosis, further disrupts the intestinal barrier and amplifies inflammation, establishing an intractable “dysbiosis—cell death—inflammation” vicious cycle (He et al., 2025). Consequently, understanding and therapeutically disrupting this cycle is paramount for the management of IBD.

## 5 Molecular mechanisms by which traditional Chinese medicine may influence PANoptosis-related pathways

Traditional Chinese medicine is characterized by multi-component, multi-target, and multi-pathway pharmacological actions. These features make TCM a plausible source of agents capable of modulating complex inflammatory cell-death networks such as PANoptosis. However, the present evidence base remains uneven. Only a limited number of studies have directly examined canonical PANoptosis markers or PANoptosome assembly. In many cases, the available data concern individual components of the PANoptosis network, such as NF-κB priming, NLRP3 activation, caspase activation, RIPK1/RIPK3 signaling, MLKL phosphorylation, mitochondrial stress, or gut microbiota-driven inflammatory stimulation. Therefore, in this review, we distinguish direct PANoptosis evidence from indirect evidence involving related cell-death or inflammatory pathways.

Among herbal monomers, baicalin currently provides relatively direct mechanistic evidence because it has been reported to inhibit mitochondrial Z-DNA formation and ZBP1-PANoptosome assembly in macrophages. Nevertheless, whether the same mechanism operates in intestinal epithelial cells, lamina propria macrophages, intestinal organoids, or IBD tissues remains to be determined. Other compounds, including berberine, matrine, and selected polysaccharides, have shown anti-inflammatory, barrier-protective, microbiota-regulatory, or apoptosis/pyroptosis/necroptosis-modulatory activities in colitis or other disease models. These findings suggest possible relevance to PANoptosis but do not yet establish direct PANoptosome inhibition in IBD.

### 5.1 Chinese herbal monomers

#### 5.1.1 Flavonoids

Among flavonoid compounds, the currently available direct evidence for modulation of PANoptosis-related machinery is limited to selected compounds and experimental contexts (Ren et al., 2025). Baicalin has been reported to interfere with mitochondrial Z-DNA formation and ZBP1-PANoptosome assembly in macrophages, providing mechanistic evidence that this compound may regulate ZBP1-associated PANoptosis under specific conditions. However, whether baicalin exerts the same effect in intestinal epithelial cells, intestinal organoids, or experimental colitis remains to be determined.

Licochalcone B has also been reported to attenuate ZBP1-dependent PANoptosis in a pulmonary fibrosis model. This finding suggests that certain flavonoids may influence ZBP1-related PANoptosis, but the evidence is derived from an extra-intestinal disease model and should not be directly extrapolated to IBD without further validation. Therefore, in the present review, licochalcone B is discussed as a hypothesis-generating example rather than as established evidence for PANoptosis inhibition in intestinal inflammation (Upmanyu and Upadhyay, 2025; Ou et al., 2026).

For other flavonoids, including quercetin, available studies more commonly address inflammatory signaling, oxidative stress, epithelial barrier protection, apoptosis, pyroptosis, or necroptosis-related markers. Unless compound-specific studies directly assess PANoptosome formation or concurrent activation of pyroptotic, apoptotic, and necroptotic pathways in intestinal models, these compounds should be regarded as potential indirect regulators rather than confirmed inhibitors of PANoptosis (Liu M. et al., 2024).

#### 5.1.2 Alkaloids

Alkaloids constitute another class of pharmacologically active constituents in TCM, among which Berberine and Matrine have drawn considerable research attention. Berberine is one of the most extensively studied alkaloids in intestinal inflammation. Existing evidence indicates that berberine can regulate inflammatory signaling, particularly NF-κB-related cytokine production, and may also influence epithelial barrier function, oxidative stress, gut microbiota composition, and cell-death-associated inflammatory responses (Wang et al., 2020). These effects are relevant to IBD because inflammatory priming through TNF-α, IFNs, TLR ligands, and NF-κB-dependent transcription can create a permissive environment for PANoptosis-related signaling (Weir and Vince, 2022; Nadendla et al., 2025). Nevertheless, the available evidence does not yet demonstrate that berberine directly inhibits PANoptosome assembly or suppresses *bona fide* PANoptosis in intestinal epithelial cells or IBD models (Mai et al., 2020; Almars et al., 2026). Therefore, berberine should be interpreted as a potential indirect modulator of PANoptosis-associated intestinal inflammation rather than as a confirmed PANoptosis inhibitor. Matrine has been reported to exert anti-inflammatory and cytoprotective effects in several inflammatory disease models, and some studies have suggested that it may influence apoptosis- or necroptosis-related signaling depending on the experimental context. However, current evidence is insufficient to conclude that matrine directly regulates PANoptosome assembly or *bona fide* PANoptosis in intestinal inflammation. For this reason, matrine is discussed here only as a candidate compound with possible relevance to cell-death crosstalk, and its role in IBD-associated PANoptosis requires direct validation using intestinal epithelial cells, organoids, and colitis models.

#### 5.1.3 Polysaccharides

Polysaccharides, such as lentinan and Ganoderma lucidum polysaccharides, are primarily derived from fungi and certain plants. Their mechanism (Qin et al., 2023)s of modulating PANoptosis are more complex and subtle, largely manifesting as protection of the intestinal microenvironment and indirect inhibition of cell death (Ishiguro et al., 2025). These polysaccharides typically do not act directly on the core proteins of the PANoptosis pathway, but rather exert their effects by reinforcing intestinal barrier function and modulating immune homeostasis. For example, they can promote mucus secretion by goblet cells, augmenting the thickness and integrity of the intestinal mucosa, thereby physically obstructing the contact of PANoptosis-inducing noxious substances with intestinal epithelial cells (Cheong et al., 2026). Meanwhile, certain polysaccharides also possess the capacity to modulate the gut microbiota, promoting the proliferation of beneficial commensals and suppressing potential pathogens, thereby reducing the stimuli that can trigger cell death at the source. Furthermore, they can modulate the function of immune cells such as macrophages, polarizing them from a pro-inflammatory to an anti-inflammatory phenotype, consequently diminishing the release of DAMPs, alleviating the “bystander effect” killing of neighboring cells, and indirectly lowering the incidence of PANoptosis (Liu et al., 2025; Zhang J. et al., 2025). The classification of cell death-related proteins is shown in Table 2.

**TABLE 2:** Representative traditional Chinese medicine-derived monomers or active components with reported or potential relevance to PANoptosis-related pathways.

Classification	Representative compound	Main reported target/pathway	Evidence/model	Interpretation for IBD/PANoptosis	Citation
Flavonoid	Baicalin	Mitochondrial Z-DNA formation; ZBP1-PANoptosome assembly	Macrophage model; direct PANoptosis-related evidence	Provides mechanistic support that baicalin can inhibit ZBP1-associated PANoptosome activation; relevance to intestinal epithelial PANoptosis in IBD remains to be validated	You et al., 2025
Flavonoid	Licochalcone B	ZBP1-dependent PANoptosis	Pulmonary fibrosis model; extra-intestinal evidence	Suggests that flavonoids may regulate ZBP1-dependent PANoptosis, but this mechanism is extrapolated to IBD and requires intestinal validation	Ren et al., 2025
Alkaloid	Berberine	NF-κB; inflammatory signaling; apoptosis/pyroptosis/necroptosis-related mediators	Mainly inflammatory and colitis-related models; direct PANoptosis evidence limited	May indirectly reduce PANoptosis-permissive inflammatory priming, but direct suppression of the PANoptosome in IBD has not been established	Reddi et al., 2021
Alkaloid	Matrine	Caspase-related apoptosis; inflammatory signaling; RIPK-related pathways	Mainly non-PANoptosis inflammatory models	Potentially relevant to cell-death crosstalk, but current evidence does not prove direct PANoptosis inhibition in IBD	-
Polysaccharide	Dendrobium officinale polysaccharide	Macrophage polarization; SENP1-SIRT3 signaling; intestinal inflammation	DSS-induced ulcerative colitis model	Supports anti-inflammatory and barrier-protective effects in colitis; direct PANoptosis regulation remains unproven	Zhang et al., 2025b
Polysaccharide	Gleditsia sinensis seed polysaccharides	Gut barrier homeostasis; gut microbiota dysbiosis	DSS-induced colitis model	May interrupt the dysbiosis–barrier injury–inflammation axis upstream of PANoptosis; direct PANoptosome inhibition remains to be tested	Qin et al., 2023

This table summarizes the mechanistic plausibility of selected traditional Chinese medicine-derived compounds in relation to PANoptosis-associated pathways. Except for compounds with direct PANoptosis-related evidence in defined experimental systems, most entries should be interpreted as indirect or hypothesis-generating. Direct evidence requires demonstration of PANoptosome assembly or coordinated activation of pyroptotic, apoptotic, and necroptotic machinery in appropriate intestinal models. Dose ranges are not listed because reported doses vary according to preparation, extraction method, route of administration, model, and experimental system.

### 5.2 Chinese herbal compound formulas

#### 5.2.1 Shaoyao Decoction

Shaoyao Decoction, derived from the Treatise on Cold Damage and Miscellaneous Diseases, is conventionally employed to treat dysentery of damp-heat pattern, with its core therapeutic actions being harmonizing qi and blood, clearing heat, and drying dampness. From a modern molecular biology perspective, Shaoyao Decoction may exert its effects by intervening at multiple key nodes within the PANoptosis pathway. The sovereign drug *Paeonia lactiflora* Pall (Paeoniaceae) and the minister drug Angelicae Sinensis Radix are rich in flavonoids and phenolic acids, constituents that can inhibit the activation of sensors such as ZBP1, thereby curbing the initiation of PANoptosis at its source (Song et al., 2024). The adjuvant drugs *Coptis chinensis* Franch (Ranunculaceae) and *Phellodendron chinense* C.K.Schneid. (Rutaceae) contain alkaloids like berberine, which can effectively suppress the RIPK1/RIPK3 signaling axis to block the necroptosis pathway, while concurrently inhibiting Caspase-1 to mitigate the pyroptotic response. More importantly, Glycyrrhizae Radix et Rhizoma in the formula serves to tonify the spleen, replenish qi, and relieve spasm and pain; its active constituents, glycyrrhizic acid and glycyrrhetinic acid, may reduce the DAMPs and PAMPs that induce cell death by modulating the gut microbiota and reinforcing the intestinal mucosal barrier, thereby diminishing the stimulation of the PANoptosis pathway (Wei et al., 2023). Therefore, the effect of Shaoyao Decoction is multifaceted: it possesses both a “hard-core” capacity for direct inhibition of key death proteins and “soft power” support achieved by ameliorating the intestinal microenvironment, forming a comprehensive, multi-layered defense system.

#### 5.2.2 Baitouweng Decoction

Baitouweng Decoction is a classic formula for treating heat-toxin dysentery, with its therapeutic actions being clearing heat, removing toxins, cooling the blood, and arresting dysentery. The core medicinals of this formula—*Pulsatilla chinensis* (Bunge) Regel (Ranunculaceae), *Coptis chinensis* Franch (Ranunculaceae), *Phellodendron chinense* C.K.Schneid. (Rutaceae), and *Fraxinus rhynchophylla Hance*. (Oleaceae)—all exhibit potent anti-inflammatory and antioxidant activities. The components of these drugs may suppress pyroptosis by downregulating the expression or inhibiting the activity of the NLRP3 inflammasome (An et al., 2022). NLRP3 is a pivotal sensor within the PANoptosis pathway, and its excessive activation represents a central step in numerous inflammatory disorders. The active constituents of Baitouweng Decoction, such as berberine and coptisine, have been substantiated to inhibit NLRP3 inflammasome assembly and activity (Zhao et al., 2025). Moreover, *Coptis chinensis* Franch (Ranunculaceae) and *Phellodendron chinense* C.K.Schneid. (Rutaceae) in the formula can suppress the NF-κB signaling pathway, thereby reducing the production of downstream pro-inflammatory cytokines (e.g., TNF-α, IL-1β) and disrupting the vicious cycle of “cell death—inflammation—more cell death”(Reddi et al., 2021). The interventional strategy of Baitouweng Decoction is thus more focused on robustly suppressing the core inflammatory axis of PANoptosis, rapidly quelling the local inflammatory storm through a radical, bottom-up approach.

#### 5.2.3 Shenling baizhu powder

Shenling Baizhu Powder epitomizes an intervention strategy centered on reinforcing the body’s vital qi and consolidating the constitution. This formula is primarily indicated for diarrhea due to spleen deficiency with dampness retention, with its therapeutic effects being replenishing qi, invigorating the spleen, and percolating dampness to arrest diarrhea. From a PANoptosis perspective, the core value of this formula lies in repairing and re-establishing the intrinsic homeostasis of the intestine. The sovereign drugs *Panax ginseng* C.A.Mey. (Araliaceae), *Atractylodes macrocephala* Koidz. (Asteraceae), and Poria possess significant immunomodulatory and nutritional support functions, which can enhance the host’s resilience and promote the repair of damaged tissues (Yi, 2025). These components may activate cell survival pathways, such as PI3K/Akt, to counteract RIPK1/RIPK3-mediated cell death signals (Wang et al., 2026). Other ingredients, including Dioscoreae Rhizoma, Lablab Semen Album, and Nelumbinis Semen, serve as excellent intestinal nourishing agents, providing essential nutrients for the regeneration of intestinal epithelial cells. Concurrently, these components may also restore the gut microecological balance by modulating the composition of the gut microbiota, consequently reducing toxic stimuli to intestinal epithelial cells at the source (Wang et al., 2025). The effect of Shenling Baizhu Powder is not to directly antagonize inflammation, but to fundamentally alter the cellular microenvironment by enhancing the body’s intrinsic “repair capacity” and “immunity,” rendering the milieu no longer permissive for the occurrence of PANoptosis. This represents a higher-level, more forward-looking intervention paradigm. The classification of cell death-related proteins is shown in Table 3.

**TABLE 3:** Representative traditional Chinese medicine formulas with potential relevance to PANoptosis-related intestinal inflammation.

Formula	Therapeutic principle	Main constituents requiring botanical standardization	Proposed mechanism relevant to IBD/PANoptosis	Evidence/model	Strength of evidence
Shaoyao decoction	Clearing heat, drying dampness, harmonizing qi and blood	Paeonia lactiflora pall.; angelica sinensis (oliv.) diels; Coptis chinensis franch.; Phellodendron spp.; Glycyrrhiza uralensis fisch. Ex DC., etc.	May reduce inflammatory priming, inhibit NF-κB-related cytokine release, support barrier repair, and regulate microbiota-derived DAMP/PAMP stimulation	Mainly colitis, inflammation, and microbiota studies	Indirect evidence for PANoptosis; direct PANoptosome inhibition remains to be tested
Baitouweng decoction	Clearing heat and detoxifying dysentery	Pulsatilla chinensis (Bunge) regel; Coptis chinensis franch.; Phellodendron spp.; fraxinus rhynchophylla hance	May reduce intestinal inflammation and microbial dysbiosis, thereby lowering upstream triggers of inflammatory cell death	Colitis and anti-inflammatory models	Indirect evidence; botanical correction made
Shenling Baizhu powder	Strengthening spleen, resolving dampness, supporting intestinal function	Panax ginseng C.A.Mey.; atractylodes macrocephala koidz.; poria cocos (schw.) wolf; dioscorea oppositifolia L., etc.	May improve epithelial barrier function, regulate microbiota, and modulate metabolic or PI3K/Akt-related survival pathways	Mainly barrier/microbiota studies; some PI3K/Akt evidence is extra-intestinal	Extrapolated evidence; direct PANoptosis evidence lacking

The table summarizes mechanistic plausibility rather than confirmed PANoptosis inhibition. Dose ranges are not listed because the reported doses vary according to preparation, extraction method, model, route, and experimental system. Future studies should report botanical authentication, voucher specimens, extraction procedures, chemical fingerprints, batch consistency, and dose justification).

## 6 Conclusion and perspective

PANoptosis provides a useful conceptual framework for understanding how pyroptosis, apoptosis, and necroptosis may converge to promote mucosal injury, epithelial barrier disruption, and inflammatory amplification in IBD. Increasing evidence suggests that PANoptosis-related molecules, including ZBP1, RIPK1, RIPK3, caspase-8, caspase-1, GSDMD, and MLKL, may participate in intestinal inflammatory responses. However, the precise contribution of PANoptosis to different intestinal cell types, disease stages, and IBD subtypes remains incompletely defined. Therefore, PANoptosis should be regarded as an emerging but still developing therapeutic concept in IBD.

Traditional Chinese medicine-derived compounds and formulas may provide valuable candidates for modulating PANoptosis-associated intestinal inflammation because of their multi-component and multi-target characteristics. These interventions may influence several upstream or parallel processes relevant to PANoptosis, including inflammatory priming, NF-κB-related cytokine production, oxidative stress, epithelial barrier dysfunction, macrophage activation, and microbiota-derived PAMP/DAMP stimulation. Such effects are biologically relevant to the intestinal inflammatory microenvironment in which PANoptosis may occur. Nevertheless, for most herbal monomers and compound formulas, current evidence supports only potential or indirect regulation of PANoptosis-related pathways rather than confirmed direct inhibition of PANoptosome assembly.

At present, the strongest direct mechanistic evidence is limited to selected compounds in specific experimental systems. For example, baicalin has been reported to inhibit mitochondrial Z-DNA formation and ZBP1-PANoptosome assembly in macrophages, while licochalcone B has been shown to regulate ZBP1-dependent PANoptosis in a pulmonary fibrosis model. These studies provide important mechanistic clues, but their relevance to intestinal epithelial cells, intestinal immune cells, and IBD pathology remains to be validated. For other compounds, such as berberine, matrine, and various polysaccharides, the available data mainly involve inflammatory signaling, barrier protection, microbiota regulation, or individual forms of regulated cell death. These findings should therefore be interpreted as hypothesis-generating rather than as evidence of established PANoptosis-targeted activity in IBD.

Classical traditional Chinese medicine formulas may also have potential value because they can act on multiple pathological processes simultaneously, including mucosal inflammation, dysbiosis, and barrier injury. However, their complex composition also creates challenges for mechanistic interpretation, botanical standardization, quality control, dose determination, and reproducibility. Future studies should use authenticated botanical materials, standardized extraction procedures, chemical fingerprinting, and clearly defined dosing strategies. More importantly, mechanistic validation should be performed in intestinal epithelial cells, intestinal organoids, DSS-, TNBS-, and IL-10-deficient colitis models, as well as human IBD tissues.

Future research should move beyond measuring isolated markers of apoptosis, pyroptosis, or necroptosis. Instead, studies should directly evaluate PANoptosome formation and the coordinated activation of key PANoptosis-related molecules, including ZBP1, RIPK1, RIPK3, caspase-8, caspase-1, GSDMD, and MLKL. Only through such rigorous validation can the field determine whether traditional Chinese medicine-derived compounds and formulas truly modulate PANoptosis in IBD, and whether these interventions can be developed into reliable PANoptosis-informed therapeutic strategies.
